# Development of Novel Management Tools for *Phortica variegata* (Diptera: Drosophilidae), Vector of the Oriental Eyeworm, *Thelazia callipaeda* (Spirurida: Thelaziidae), in Europe

**DOI:** 10.1093/jme/tjab171

**Published:** 2021-11-08

**Authors:** M A González, D Bravo-Barriga, P M Alarcón-Elbal, J M Álvarez-Calero, C Quero, M Ferraguti, S López

**Affiliations:** 1 Institute of Tropical Medicine and Global Health (IMTSAG), Universidad Iberoamericana (UNIBE), Avenida Francia 129, 10203, Santo Domingo, Dominican Republic; 2 Universidad de Extremadura, Facultad de Veterinaria, Departamento de Sanidad Animal, Parasitología, Avda. Universidad s/n, 10003 Cáceres, España; 3 Laboratorio de Entomología, Universidad Agroforestal Fernando Arturo de Meriño (UAFAM), 41000, Jarabacoa, Dominican Republic; 4 Department of Biological Chemistry, Institute for Advanced Chemistry of Catalonia (IQAC-CSIC), Jordi Girona 18-26, 08034 Barcelona, Spain; 5 Department of Theoretical and Computational Ecology (TCE), Institute for Biodiversity and Ecosystem Dynamics (IBED), University of Amsterdam, Science Park 904, 1098XH Amsterdam, The Netherlands

**Keywords:** zoophilic fruit fly, field test, color, netting, bait

## Abstract

Lachryphagous males of *Phortica variegata* (Fallén, 1823) are gaining increasing attention in Europe, as they act as vectors of the nematode *Thelazia callipaeda* Railliet & Henry, 1910, causal agent of thelaziosis, an emergent zoonotic disease. Currently, there are no effective control strategies against the vector, and surveillance and monitoring rely on time-consuming and nonselective sampling methods. Our aim was to improve the knowledge about the population dynamics and the chemical ecology of the species. A total of 5,726 *P. variegata* flies (96.4% males and 3.6% females, mostly gravid) were collected in field experiments during June–September of 2020 in an oak forest in northern Spain. Our results indicate that 1) by means of sweep netting a significantly higher number of captures were found both around the collector´s body and in the air than at ground level; 2) a positive relationship was detected between the abundance of *Phortica* flies and temperature, with two significant peaks of abundance at 24 and 33°C; 3) the blend of red wine and cider vinegar was the most attractive bait; 4) yellow traps captured fewer flies compared to black and transparent traps; and 5) a significant reduction toward vinegar and wine was detected in presence of the phenolic monoterpenoid carvacrol. In addition, all the males (*n* = 690) analyzed by both molecular detection and dissection resulted negative for the presence of *T. callipaeda* larvae. Overall, these findings provide a better understanding of the vector in terms of monitoring and management strategies.

The number of emerging and reemerging infectious diseases affecting humans is currently increasing (Cupertino et al. 2020), and approximately 75% of these diseases are known to be of zoonotic origin (Vorou et al. 2007). Regardless of arboviruses, emerging parasitic infestations are gaining relevance. Although protozoa are more likely to be responsible for most of them, several metazoan infestations have raised the alarm in recent times as major public health concerns (Weiss 2008).

Accordingly, the oriental eyeworm, *Thelazia callipaeda* Railliet & Henry, 1910, causal agent of the vector-borne zoonotic disease thelaziosis that causes different ocular lesions with variable degree of severity (including conjunctivitis, keratitis, corneal opacity, ulcers, and even blindness), has gained great attention in Europe over the last decades (Do Vale et al. 2020, Otranto et al. 2021). After the detection of the first case of canine thelaziosis in Italy in the late 1980s (Rossi and Bertaglia 1989), as well as in foxes and cats (Otranto et al. 2003), the disease has spread throughout almost all mainland Europe, not only favored by pet trade (Graham-Brown et al. 2017, Silva et al. 2020), but also by the emergence of autochthonous foci with high infection rates (Marino et al. 2020). In addition to Europe, a recent case report of autochthonous canine thelaziosis in the United States has arisen great concern among medical and veterinary communities, and evidences the spread of the parasite overseas (Schwartz et al. 2021). Phylogenetic analyses of extirpated eyeworms revealed that they belong to the European haplotype-1 (Schwartz et al. 2021), which would imply a possible introduction of *T. callipaeda* from abroad, and in turn reinforces the fact that American populations of *P. variegata* are competent vectors for this nematode (Otranto et al. 2018). To date, *T. callipaeda* infections in Europe have been reported in wild animals (e.g., red fox, wolf, beech marten, and badger; Otranto et al. 2009, Ionică et al. 2018, Seixas et al. 2018), domestic carnivores (dogs and cats; Otranto et al. 2003, Dorchies et al. 2007, Maia et al. 2016), and lagomorphs (hares and rabbits; Otranto et al. 2009). On the other hand, the number of cases of *T. callipeda* infection in humans has increased during the last 10 yr in numerous European countries (Otranto et al. 2013), such as those reported in Italy and France (Otranto and Dutto 2008), Spain (Marino et al. 2020), and more recently in Croatia, Serbia, and Germany (Paradžik et al. 2016, Tasić-Otašević et al. 2016, Dolff et al. 2020).

In Spain, the first autochthonous case of thelaziosis was reported in 2010 in a dog from the Cáceres province (Miró et al. 2011). Shortly thereafter, the first human case of thelaziosis was reported in the same province (Fuentes et al. 2012). Recent findings indicate new areas of central Spain have to be considered as autochthonous for canine thelaziosis (Marino et al. 2018), which reaffirms its status as an emerging zoonotic disease that deserves public awareness and knowledge (Otranto and Dutto 2008).

In Europe, the fly species *Phortica variegata* (Fallén, 1823) has been proved to be involved in the transmission of the parasite both under experimental and natural conditions (Otranto et al. 2005, 2006b). Male adults of *P. variegata* act as the intermediate host of the parasite, and due to their tear-feeding behavior, the parasite spreads from one to another host. Males feed on lachrymal secretions of infested animals during summer, and while feeding, first-stage larvae are ingested. After developing to the third-stage inside the fly, they are transmitted when encountering a new suitable host (Otranto et al. 2005). In addition to parasite transmission, *P. variegata* activity also represents a serious nuisance to host, especially for humans, due to its hovering activity while seeking the eyes and body transpiration (Otranto et al. 2006a). The distribution range of this fly is related to reports of cases of thelaziosis in Europe (Do Vale et al. 2020), i.e., regions at 800–1,000 m.a.s.l. characterized by a continental Mediterranean climate, with deciduous forests (mainly *Quercu*s spp.), and the presence of nearby rivers (Otranto et al. 2006a, Palfreyman et al. 2018). Based on an ecological niche modeling, the northern Spain is suggested as a suitable area for the development of this zoophilic fly species (Palfreyman et al. 2018). Despite some infection cases detected in dogs and cats (Marino et al. 2020), and the occurrence of naturally infested *P. variegata* males in central Spain (Marino et al. 2018), no information is available on the prevalence of *T. callipaeda* on *P. variegata* populations in northern Spain. Under this context, the zoonotic nature of *T. callipaeda* and its tight relationship with *P. variegata* strongly promote the need to develop effective vector-targeted control methods.

From an integrated pest management perspective, a bait-based mass trapping strategy could be considered as a feasible eco-friendly approach. So far, *P. variegata* surveillance and monitoring rely on two broadly different sampling methods that result in a skewed sex ratio. On the one hand, direct netting around the host eyes collects mainly males (Roggero et al. 2010, Marino et al. 2018), while the sex ratio from fruit-baited traps containing sliced pieces of fruits (e.g., apples, bananas, and pears), tends to be more balanced, with large fluctuations depending on the sampling season (Otranto et al. 2006a, Roggero et al. 2010). In addition, it is suggested that beer traps hung in the tree canopy may be attractive for females (Máca 1977). However, none of these methods should be considered as a feasible alternative for mass trapping, due to their inherent limitations, such as time loss and host dependency (net sweeping; Marino et al. 2018), and low specificity (fruit baits; Roggero et al. 2010). Consequently, the development of a more selective, effective, and user-friendly trapping system would represent a more useful tool for surveillance, monitoring, and even mass trapping of this pest species.

Our aim was to assess in field trials, the abundance of *P. variegata* flies with respect to different heights and climatic characteristics (temperature and wind); the attractiveness of different fermented baits compared to that exerted by fruit bait; the influence of different trap colors as a visual stimulus; the repellence exerted by different monoterpenoids; and the detection of *T. callipaeda* in feral males. Overall, these findings will represent a first step in shedding light on different factors that may interact mediating the attraction of *P. variegata*, contributing thus to a better understanding of potential management strategies for this vector species.

## Material and Methods

### Study Area and Insect Sampling, Processing, and Taxonomic Identification

**Fig. 1.**
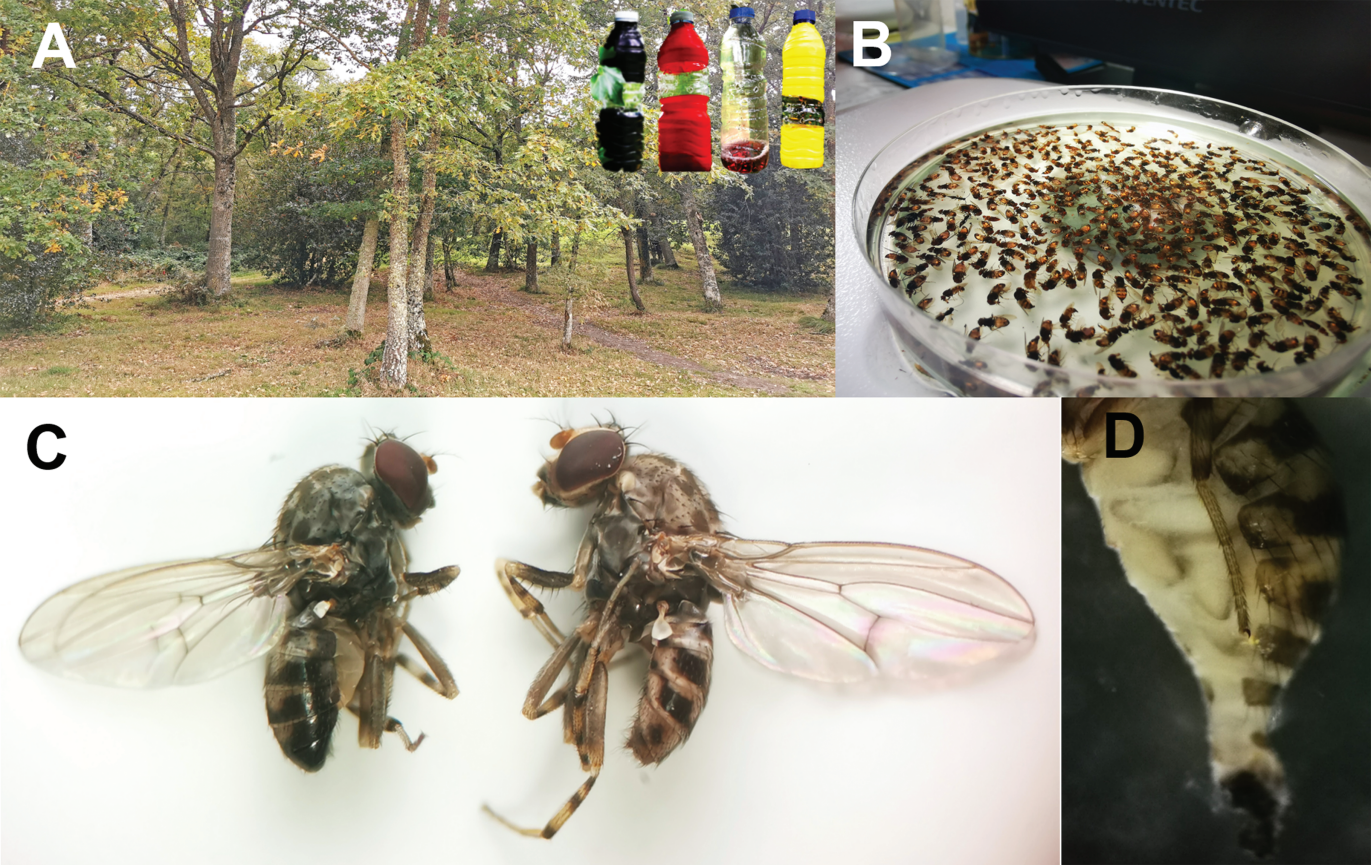
Sampling site composed of *Quercus pyrenaica*, and inset of the color traps (from left to right: black, red, transparent, and yellow) used in Experiment C (top right; A). Specimens of *Phortica variegata* in ethanol (70%; B). Lateral view of male (left) and female (right) of *P. variegata*, respectively (C). Lateral view of the abdomen of a gravid female (D).

Field trappings were conducted in a private recreational area (ca. 75 ha; 42.660420, −2.510518; altitude 745 m.a.s.l.) with high human attendance and serious nuisance repeatedly caused by *P. variegata* over the last summers. The sampling area was located within the Izki Natural Park (ca. 9,150 ha), a forested area at the eastern border of the province of Álava (Basque Country, northern Spain), characterized by cold, rainy winters and hot, dry summers. The average mean temperature, total precipitation, and relative humidity during the sampling period was 16.7°C (max mean 24.3°C, min mean 9.8°C), 171 l/m^3^, and 70.8%, respectively (data from a weather station located ca. 4.4 km from the sampling points, accessed through www.euskalmet.com). The flora is dominated by the Pyrenean oak (*Quercus pyrenaica*; Fig. 1A), constituting one of the largest oak woodlands (ca. 3,500 ha) in Europe. Additionally, *Quercus robur*, *Ilex aquifolium*, *Fagus sylvatica,* and *Prunus* sp. are also relatively abundant in the area.

All the insects trapped were preserved in ethanol (70%) and stored at −20°C until further morphological identification under a stereomicroscope (4×; Digilab, model DI-224, Bethesda, MD; Fig. 1B). *Phortica variegata* individuals were segregated from by-catch according to diagnostic species-specific features (Máca 1977) and sorted by collection date. Subsequent gender determination was based on the morphology of the terminal part of the abdomen, i.e., when pressing the abdomen females extend the ovipositor, whereas males have a visible aedeagus. In addition, the body of females tends to be paler than in males (Fig. 1C; Otranto et al. 2006a). Gravid females were also noted by the presence of eggs in the abdomen (Fig. 1D).

### Experiment A: Assessment of Different Net Sweeping Heights and Climatic Conditions on *P. variegata* Abundance

Sampling was conducted from 01-VI-20 to 01-VIII-20, by sweep netting with a standard entomological polyester net to assess the spatial distribution of the flies at three different heights: at ground level (on grass and shrubs), on human (around the face of the collector, ca. 175 cm), and at a height of 200–220 cm above the ground (below the tree canopy and open areas in the landscape). A total of 15 sampling transects were conducted over a 3-mo sampling period (5 per month) between 16:00 and 18:00 h, as preliminary observations revealed that *P. variegata* is active throughout these hours. The transect, consisting of shaded and sunny areas, was zig-zagged over a distance of 150 m with three stops of 10 s every 50 m by the same collector, to avoid any bias in sweep-net samples (Cooper et al. 2012). An average of 60 strokes per sampling height were performed per transect, one after the other, with a maximum of 5 min for emptying the contents between each sweeping substrate. Captured flies were aspirated with a mechanical aspirator (InsectaVac aspirator, BioQuip Products Inc., Compton, CA), and stored in ethanol (70%) until further identification. To correlate data collection with the influence of climatic factors, temperature (°C), and wind speed (m/s) were recorded daily at the beginning and at the end of sample collection using an anemometer (Proster TL0017, Hong Kong, China).

### Experiments B–D: General Methodology

A total of three consecutive field trapping experiments (encoded hereafter as Experiments B, C, and D) were deployed from 01-VI-20 to 30-IX-20, when the flight activity of *P. variegata* peaks (Marino et al. 2018). Each experiment contained from three (3 × 3 Latin square design; Experiment B) to four traps (4 × 4 Latin square design; Experiments C and D), placed at least 15 m apart from one to each other. Traps operated for three consecutive days within an experiment, after which the trap was emptied and randomly rotated to a new position. Each experiment lasted 24 d, accounting hence for a total of 8 replicates. For trap selection, the methodology of Roggero et al. (2010) was adapted, using transparent plastic bottles (330 ml) drilled with 14 holes (4 mm diameter) randomly distributed along the medium height of the bottle. Bottle traps were vertically suspended of an oak tree at 1.60−1.80 m above the ground level. New bottles were used after each trap collection. Before any trial, empty bottles were tested for three consecutive days as blank controls, to check whether *P. variegata* entered the trap in absence of any bait. Details of each experiment are provided separately below.

### Experiment B: Assessment of the Attraction of Different Baits on *P. variegata*

Three different baits were selected to be tested, according to their attractiveness on *P. variegata* (Otranto et al. 2006b, Palfreyman et al. 2018), and drosophilid species (Landolt et al. 2012, Iglesias et al. 2014). Each bait consisted of 1) a slurry mixture of rotten fruits (45 g of banana, apple, and pear) mixed with 5 ml of water, 2) an admixture (50 ml) of cider vinegar (75%) and red wine (25%), and 3) activated yeast in powder (10 mg) and sugar (10 g) diluted in water (30 ml). The mixture of rotten fruits was prepared in bulk, stored at −20°C, and left at room temperature for 12 h before being deployed in field. Each bait was renewed on each trap collection day.

### Experiment C: Assessment of the Influence of Trap Color on *P. variegata* Catches

The efficiency of three different colored bottle traps (black, red, and yellow) was tested against that of the same transparent bottle previously used in Experiment B (Fig. 1A). For obtaining the colored bottles, transparent bottles were completely lined with an adhesive tape of the corresponding color. The mixture of red wine and cider vinegar (50 ml) was used as standard bait for each treatment, as it proved to be the most effective attractant in Experiment B.

### Experiment D: Assessment of the Potential Repellency of Different Terpenoids on *P. variegata*

Trial and error assays showed that 10 ml of the blend of red wine and cider vinegar was the minimum optimal amount to attract flies to trap bottles. Thereby, three monoterpenoid compounds, namely 2-isopropyl-5-methylphenol (hereafter referred as thymol), 5-isopropyl-2-methylphenol (carvacrol), and 1,8-cineole (eucalyptol), were tested as potential disruptants of the bait attractiveness, given their proven repellency on different insect groups (Evergetis et al. 2018, Oliveira et al. 2018, da Silva and Ricci-Júnior 2020, Lee et al. 2020). Carvacrol (98%) and eucalyptol (>97%) were obtained from Merck/Sigma–Aldrich (Madrid, Spain), while thymol (>98%) was acquired from Alfa Aesar (Karlsruhe, Germany). Two different experiments (referred hereafter as Experiment D1 and D2) were designed. In Experiment D1, Ziploc bags (5.5 × 3.5 cm) were used as dispensers, and 1 ml of each compound was loaded onto a cellulose pad (3.5 cm × 2.5 cm) that was enclosed in the Ziploc bags. This type of dispenser allowed to obtain an approximate release rate for each compound as follows: thymol 7 mg/d, carvacrol 3 mg/d, and eucalyptol 59 mg/d. In Experiment D2, the same Ziploc bags were used as dispensers, although in this case 1 ml of the corresponding compound was loaded in a cotton pad (3.5 cm × 2.5 cm), providing approximately the following release rates: thymol 15 mg/d, carvacrol 5 mg/d, and eucalyptol 75 mg/d. Release rates were estimated based on daily weight loss during 2 wk under laboratory conditions before the deployment of each experiment. In both experiments, the corresponding dispenser was hung from the inner top of the bottle trap by using a wire, so that it was aligned with the drilled holes of the bottle walls.

### Detection of *T. callipaeda* in *P. variegata* Males by Dissection and Molecular Analysis

A total of 300 males of *P. variegata* obtained during Experiment A were examined for the presence of *T. callipaeda* L3 larvae. First, live males were taken to the laboratory, dissected under the stereo microscope (4–8×) on a Petri dish with a drop of physiological saline solution, and visually checked for the presence of the nematode in the proboscis.

Second, a total of 390 randomly chosen *P. variegata* males from Experiments B–D were grouped by collection date and pooled (15 individuals/pool) in vials with ethanol (70%) at −20°C for further molecular analysis. Genomic DNA was extracted from pools using a QIAamp DNA Mini Kit (QIAGEN GmbH, Hilden, Germany) according to the manufacturer’s instructions. Total DNA was purified using the QIAamp DNA Mini Kit (Qiagen, Germany) and stored at −20°C. The *cox1* gene was partially amplified using the primer set COIintF (5’-TGATTGGTGGTTTTGGTAA-3’) and COIintR (5’-ATAAGTACGAGTATCAATATC-3’) following PCR protocol as described previously for Spirurida (Casiraghi et al. 2001). The amplified products of approximately 689 bp were analyzed by electrophoresis in 1.5% agarose gels stained with Green Safe Premium (Nzytech, Portugal), using a 100-bp DNA ladder as a molecular weight marker and observed under UV light.

### Statistical Analyses

The effect of sampling height (considered as a categorical independent variable) and climatic variables (i.e., temperature and wind, continuous independent variables) on the *P. variegata* abundance (mean number of flies captures, continuous dependent variable) collected by net sweeping were analyzed using linear mixed-effects models (LMM) fitted by maximum likelihood with Gaussian distribution. The number of insect captures was log-transformed to normalize its distribution. Trapping site was included as random factors to account for the geographical variation of the sampling design.

In addition, to explore the relationship between environmental explanatory variables and *P. variegata* abundance, the nonparametric algorithm Random Forest (RF) regression analyses based on 1,000 trees was used (Breiman 2001). This is a machine-learning algorithm increasingly being used in ecology during recent years (Cutler et al. 2007, Fox et al. 2017, Brieuc et al. 2018), which makes it possible to identify nonlinear relationships that would otherwise be impossible to trace. The most important variables selected in the model were listed following the percentage of increase in Mean Square Error (%IncMSE) splitting criterion to find the optimal predictors.

On the other hand, the effect of different treatments (i.e., attractive blends, color, and monoterpenoid compound), on the collection of *P. variegata* in experiments B, C, and D, were analyzed using generalized linear mixed models (GLMM). In each experiment, insect captures per each treatment were included as dependent variable, while bait, trap color, and monoterpenoid compound were set as categorical independent factors. Separate models were performed for each treatment with a negative binomial error distribution and logarithmic link, rather than Poisson distributed error, to reduce model overdispersion caused by the aggregation of captures (Ver Hoef and Boveng 2007, Roiz et al. 2012).

The collinearity between independent variables was tested for each model using the variance inflation factor (VIF; Zuur et al. 2010), and residuals were checked for both the *qq-plots* and the composite hypothesis of normality with Lilliefors (Kolmogorov–Smirnov) test, due to sampling size was higher than 50 records (Thode 2002). In addition, GLMM overdispersion was controlled using Pearson statistics (ratio of the Pearson χ ^2^ to its degrees of freedom), a common method used for assessing the deviance of goodness-of-fit statistics (Rodríguez 2001). There was no evidence of collinearity between the variables included in the models nor overdispersion, as the Pearson dispersion statistics were always close to 1. The ANOVA analysis of variance was used to calculate the χ ^2^, and post hoc analyses were performed using Tukey contrasts test when significant differences were detected (*P* ≤ 0.05).

All statistical analyses were conducted in R (v3.6.3; The R Foundation for Statistical Computing Platform 2020) using the following packages: *arm*, *car*, *lme4*, *MASS*, *Matrix*, *MuMIn*, *multcomp*, *randomForest*, *Rcpp*, and *stats*. Partial dependence plot was developed for showing nonlinear relationships of the predictor variables.

## Results

Overall, 5,726 *P. variegata* flies (96.4% males and 3.6% females) were collected during the summer of 2020 (Experiment A = 247 males and 5 females, Experiment B = 944 males and 5 females, Experiment C = 997 males and 28 females, and Experiment D1 and D2 = 3,051 males and 157 females, and 281 males and 11 females, respectively). The percentage of females captured differed depending on the month as follows: June (0%), July (0.8%), August (4.2%), and September (16.6%). Up to 86% of the females were gravid.

### Experiment A

**Fig. 2.**
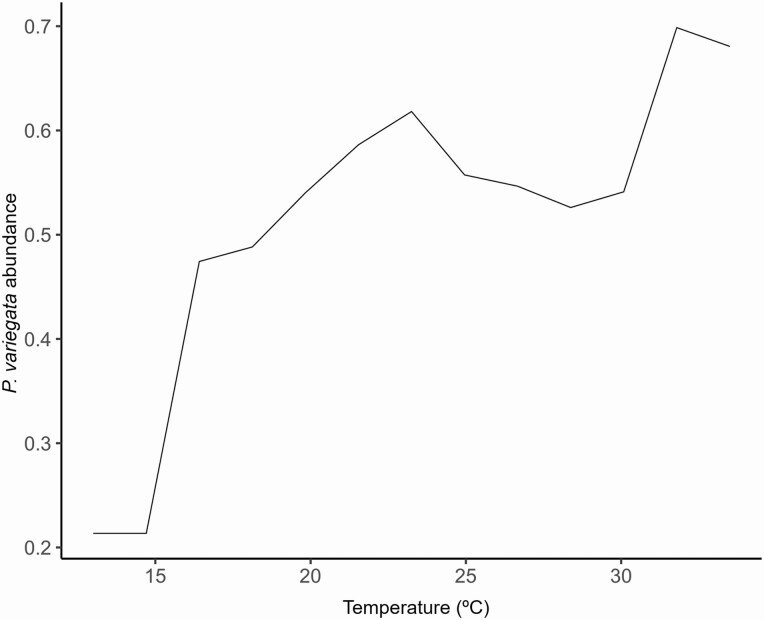
Partial dependence plot for the RF analyses between *Phortica variegata* abundance (log-transformed) and the temperature (°C) of each sampling date (*n* = 247).

A total of 252 individuals of *P. variegata* flies were captured by net sweeping at three different heights, showing an overall pronounced male-biased sex ratio (98%). Those collected around the human body accounted for 56%, followed by 40% gathered below the canopy (200–220 cm from ground) and only 4% at ground level. Overall, the explained variance for the *P. variegata* abundance LMM was 35.5% (variance of the predictions divided by the variance of the response). Briefly, significantly more captures were found both around the collector´s body (Tukey post hoc test, estimate ± SE = 0.579 ± 0.166, df = 37, *z* = 3.498, *P* = 0.001) and below the canopy (estimate ± SE = 0.057 ± 0.165, df = 37, *z* = 3.446, *P* = 0.002) than at ground level. Also, a marginally significant positive relationship between insect abundance and the temperature (estimate ± SE = 0.022 ± 0.011, df = 37, *t* = 2.010, *P* = 0.051) was found. Here, the partial plot evidenced by the RF showed an increasing nonlinear trend between the abundance of the flies and temperature, with two peaks at 24 and 33°C (Fig. 2). The minimum daily temperature recorded with catches was 13.0°C and the maximum 33.5°C.

### Experiment B

**Fig. 3.**
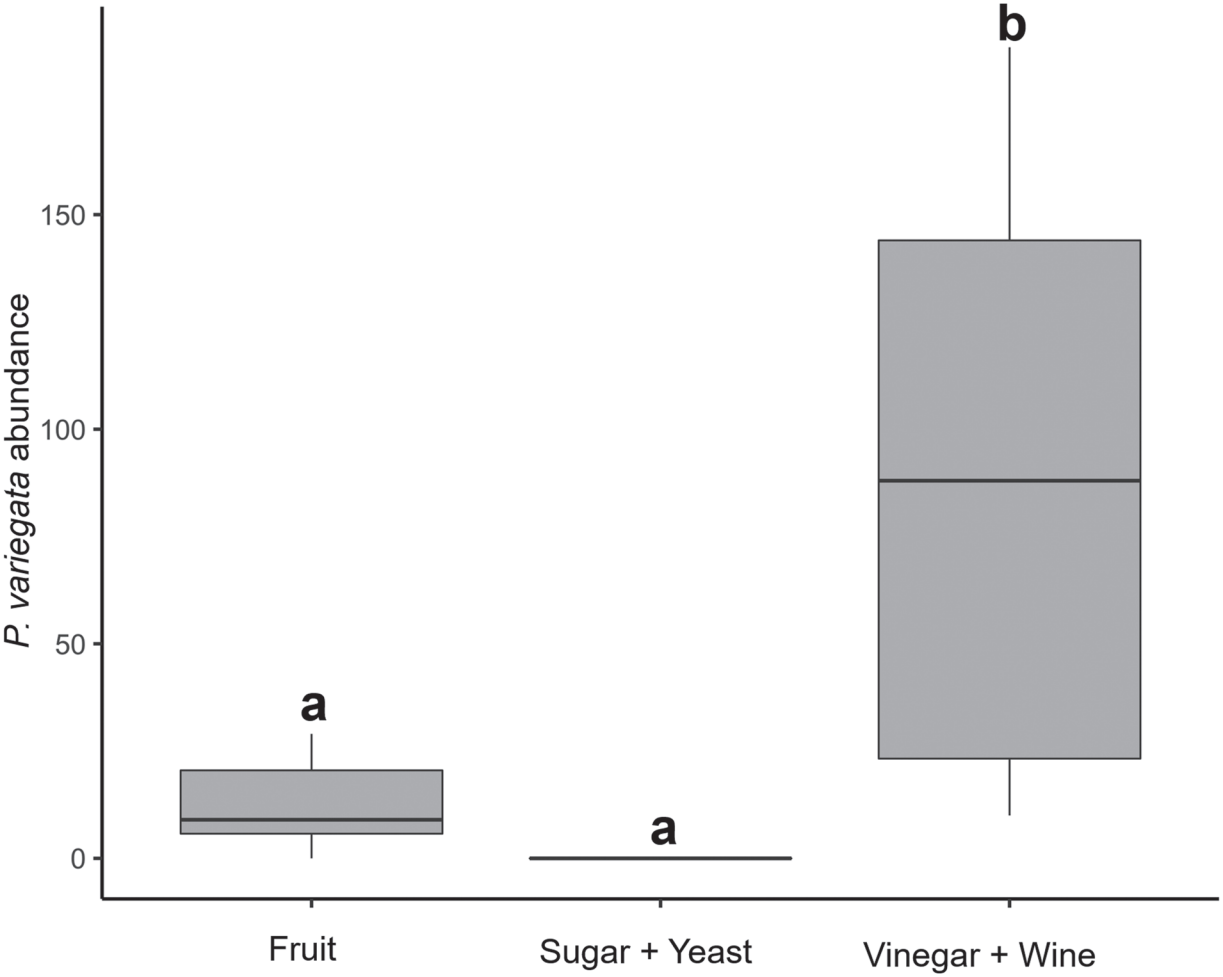
Box-and-whiskers plots of the number of *Phortica variegata* catches (log-transformed) in traps with different baits. Boxes with different superscript letters are statistically different (*P* ≤ 0.05).

A total of 949 *P. variegata* flies were caught in this trial. Significant differences were found in the attractiveness of the different baits (χ ^2^ = 16.034, df = 2, *P* ≤ 0.001). The binary blend of red wine and cider vinegar was significantly more attractive to flies than the mixture of fruits (Tukey post hoc test, estimate ± SE = 1.954 ± 0.747, *z* = 2.615, *P* = 0.024) and yeast plus sugar (estimate ± SE = 2.976 ± 0.759, *z* = 3.920, *P* ≤ 0.001; Fig. 3). No significant differences were recorded between the mixture of fruits and yeast plus sugar. In terms of specificity, the binary blend yielded the best results, with the least number of nontarget insects (mainly *Drosophila* spp.) trapped (12%), whereas the mixture of fruits and yeast plus sugar accounted for 68% and 28% of drosophilid by-catch, respectively.

### Experiment C

**Fig. 4.**
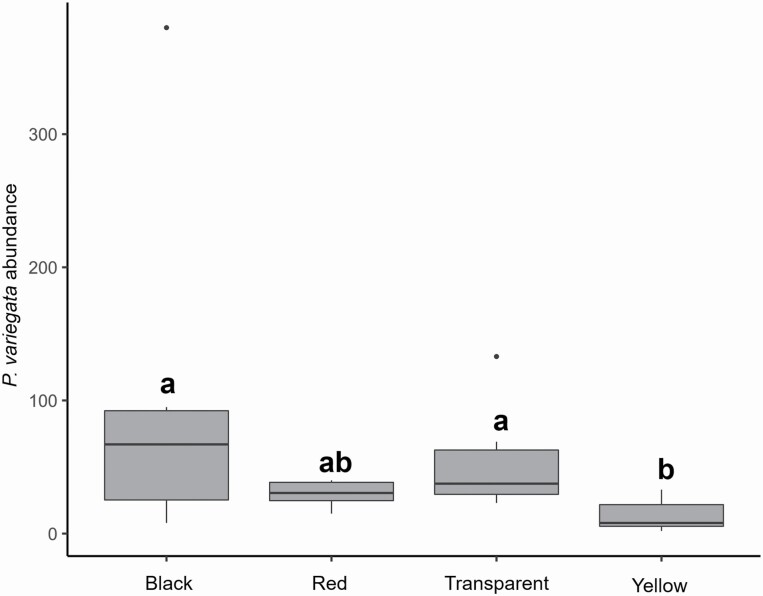
Box-and-whiskers plots of the number of *Phortica variegata* catches (log-transformed) in trap bottles of different colors baited with the binary blend of cider vinegar and red wine (50 ml). Boxes with different superscript letters are statistically different (*P* ≤ 0.05).

A total of 1,025 *P. variegata* flies were trapped in the experiment, varying significantly according to the trap color (*χ*^*2*^ = 22.241, df = 3, *P* = <0.001). Yellow baited-traps captured fewer flies than both black (Tukey post hoc test, estimate ± SE = −1.909 ± 0.4224, *z* = −4.519, *P* = <0.001) and transparent baited-traps (estimate ± SE = −1.451 ± 0.425, *z* = −3.411, *P* = 0.004; Fig. 4). In contrast, no significant differences were detected between the number of caches found in red, black, and transparent traps (Fig. 4).

### Experiment D

**Fig. 5.**
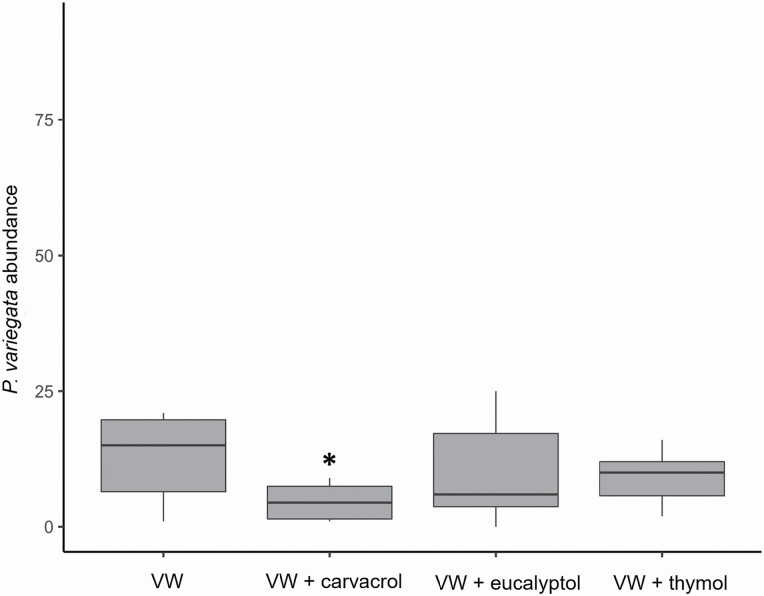
Box-and-whiskers plots of the number of *Phortica variegata* catches (log-transformed) in traps baited with 10 ml of the binary blend of cider vinegar and red wine (VW), and the combination of VW and carvacrol (5 mg/d), eucalyptol (75 mg/d), or thymol (15 mg/d). Asterisk denotes statistical differences between treatments and control (*P* ≤ 0.05).

A total of 3,208 and 292 *P. variegata* flies were collected in the Experiments D1 (low release rate) and D2 (high release rate), respectively. No significant reduction in trap catches was found for any of the monoterpenoids in Experiment D1. Conversely, a significant reduction in the number of catches was observed in Experiment D2 (*χ*^*2*^ = 9.347, df = 3, *P* = 0.025). Traps baited with carvacrol (release rate ca. 5 mg/d) significantly collected fewer *P. variegata* flies compared to control traps (Tukey post hoc test, estimate ± SE = −1.665 ± 0.552, *z* = −3.014, *P* = 0.014; Fig. 5). No significant differences were found for traps releasing thymol and eucalyptol in comparison to the control trap (Fig. 5).

### Detection of *T. callipaeda*

None of the 690 *P. variegata* males subjected to dissection (*n* = 300) and molecular analysis (*n* = 390) resulted positive in the detection of *T. callipaeda* larvae.

## Discussion

Our field sampling revealed the abundant presence of *P. variegata* in a *Q. pyrenaica* forest in northern Spain, in agreement with previous works that highlight deciduous woods with continental Mediterranean climate as a common ecological niche for this species (Otranto et al. 2006a, Palfreyman et al. 2018). Unfortunately, the resting places of this species within this habitat remain still unknown, although Phortica spp. are predominantly dwelling in the tree canopy (Toda 1987, Máca and Otranto 2014) and trunks (Chen et al. 2006). Net sweeping showed that *P*. *variegata* rarely rests on vegetation at ground level, and both aerial sweeping and around the eyes of the collector were efficient methods to collect flying adults. The relative high number of aerial collections might indicate that a reasonable number of flies were flying in the sunny and shade areas within the oak grove, most likely patrolling along the forest tracks, as reported by Máca and Otranto (2014). Among the climatic features considered, temperature was demonstrated to have a significant influence on the activity of the species, as revealed by our sweep netting data. In fact, a positive correlation between the number of catches and the mean temperature was recorded. The role of temperature as an important driver of *P. variegata* abundance has been recently addressed by Pombi et al. (2020), who detected a direct correlation between temperature and population dynamics of *P. variegata* in different countries. Our observations indicate that these flies start flying at 18°C and temperatures above, as almost no flies were captured at lower temperatures. Indeed, temperature drops below 16°C interrupt the activity of the fly, and lower than 10°C provokes a sharp cease of activity (Otranto et al. 2006a). Nonetheless, and although overwintering is known to occur in *P. variegata* (Otranto et al. 2005), those environmental factors that may be responsible of triggering the end of this diapausing period have not been determined yet. Additional climatic variables that may be correlated with the abundance of males, such as relative humidity and barometric pressure (Pombi et al. 2020), were not taken into consideration in our study.

Field trapping collections revealed that the activity of *P. variegata* males in the Basque Country expands from at least June until September. Interestingly, our collections were strongly male-biased throughout the trapping period, regardless of the sampling method employed. However, a marked increase in the proportion of females was observed at the end of summer. It is well known that males of *P. variegata* are predominant in flying around the eyes of the host (Otranto et al. 2006a). However, the ratio of females to males is still a controversial issue, with differing findings depending on the sampling methodology and even season. For instance, remarkable changes in sex ratio have been observed when netting around host eyes and fruit baits in Italy, and they seemed to vary widely according to seasonality (Otranto et al. 2006a, b). Fly collections in southern Italy revealed that, while females were predominant from May to June, the prevalence of males increased in August, drastically biasing to a male:female ratio of 181:1 by September–October (Otranto et al. 2006b). However, Roggero et al. (2010) observed an overall predominance of females regardless of the season in Switzerland. In our field trials, males prevailed during the whole sampling period (i.e., from June to September), albeit, it is also worth noting that a high percentage of trapped females with the binary blend of cider vinegar and red wine was reported as gravid from July to September. We suggest that reproductive and/or mating status may be modulating the attraction towards this bait, as reported for other drosophilids (Matsunaga et al. 1995, Wong et al. 2018, Clymans et al. 2019). For instance, in other Steganinae species, females and immature males are attracted to fruit baits, while reproductively mature males are not (Matsunaga et al. 1995). Additionally, nongravid females of the major crop pest *Drosophila suzukii* (Diptera: Drosophilidae), are more attracted to fermented-based fruits (Wong et al. 2018). In contrast to one would expect, gravid females were attracted to fermented baits in our study. Taken into consideration that natural oviposition substrates of *P. variegata* are still unknown, and their ability to lay eggs on fresh fruit under artificial conditions (Otranto et al. 2012), our findings suggest that protein intake from this kind of baits may be of an undetermined relevance for the physiology of egg-bearing females. Therefore, a long-term monitoring of populations would allow to infer the factors mediating these shifts in population dynamics, which in turn may shed light on the ecological implications underlying the attraction on both sexes of *P. variegata* mediated by the binary blend of vinegar and wine.

Our outcomes have demonstrated for the first time the suitability of red wine and cider vinegar as a cost-effective approach for trapping *P. variegata.* The binary blend proved to be more attractive and selective when compared to traditional fruit-based baits (Roggero et al. 2010). This blend has previously been reported to be highly attractive to drosophilid species, such as *D. suzukii* and the African fig fly *Zaprionus indianus* (Landolt et al. 2012, Cha et al. 2015a). Another congeneric species, *Phortica semivirgo*, has previously been found in traps filled with red wine and apple vinegar in Hungary (Kerezsi et al. 2019). In *Phortica* spp., lachryphagy is considered to be the main zoophilic feeding habit, although they are also often found feeding on fruits, fermenting substrates, and sap fluxes of tree trunks (Chen et al. 2006, Otranto et al. 2006b, Bächli et al. 2008, Máca and Otranto 2014). In contrast, some genera of Steganinae are known to be rarely attracted to beer or fruit baits, such as *Cacoxenus, Stegana, Gitona*, and *Leucophenga* (Otranto et al. 2008). This attraction towards fermented cues is in concordance to some extent with many species within the subfamily Drosophilinae, which find rotten and decaying substrates as suitable for breeding and/or feeding. Indeed, fermentation-related volatiles have been demonstrated to play a pivotal role as olfactory cues mediating attraction (Becher et al. 2010, 2012; Kleiber et al. 2014; Cha et al. 2015a), and therefore the use of different fermented products-releasing baits have been widespread used for drosophilid trapping (West 1961, Bächli et al. 2008, Cooper et al. 2012, Landolt et al. 2012, Epsky et al. 2014, Iglesias et al. 2014, Hottel et al. 2015, Cruz-Esteban et al. 2021). Thus, our results provide a robust basis for considering the binary blend as a promising tool for monitoring and/or mass trapping of *P. variegata*, albeit a simplification of this mixture into a multicomponent bait may represent a more selective attractant (Cha et al. 2015b). Therefore, further studies on the olfactory and behavioral response of male *P. variegata* are needed to elucidate which key active volatiles in the binary mixture are responsible for mediating the attraction to improve the current blend.

The orientation of *P. variegata* males toward a colored stimulus has not been addressed so far. According to our results, the color of the trap does not appear to be a key factor in the attraction to the trap when combined with the binary mixture of wine and vinegar. Three of the colored visual stimuli (i.e., transparent, black, and red) performed similarly, with only the yellow color showing a negative effect on captures. This aversive behavior was also observed for yellow sticky traps placed on trees (data not shown). The influence of yellow color in the number of catches has also been noticed for some Drosophilidae. In the case of *D. suzukii*, different studies have pointed out that the avoidance or preference for yellow color depends on the bait type (Iglesias et al. 2014, Bolton et al. 2021). In this regard, the number of *D. suzukii* found in yellow traps increases synergistically with the leaf volatile β-cyclocitral, whilst the preference for this color is reduced in presence of host or yeast-odors (Bolton et al. 2021). Similarly, Iglesias et al. (2014) observed that catches of yellow traps baited with apple cider vinegar did not increase. However, yellow and yellow-green cards are the most attractive stimuli for *Z. indianus* when deployed with a commercial bait (Cruz-Esteban et al. 2021). In the case of *P. variegata* males, the negative effect of yellow color might be related to the host seeking behavior of the species, as observed for other flies. For example, in the case of horse flies and deer flies (Diptera: Tabanidae), yellow and green-colored trapping systems have been demonstrated to be less attractive for females than, for instance, black and red colors (Bracken et al. 1962, Allan and Stoffolano 1986). This behavior has been associated to the lack of contrast of the target with regard to the surrounding background, a relevant fact that it is certainly related to host location and orientation (Allan and Stoffolano 1986). In a similar vein, although those stimuli governing the orientation of *P. variegata* males to hosts are still unknown, we suggest that visual cues may be relevant in long-range host seeking.

To our knowledge, no research based on repellency studies on *P. variegata* has been published. Among the monoterpenoids evaluated, only carvacrol significantly reduced the number of male captures at the highest release rate (ca. 5 mg/d). Previous studies have demonstrated the repellency of this compound on arthropods of medical-veterinary interest (Park et al. 2005, Masoumi et al. 2016, Evergetis et al. 2018, Lima et al. 2019) and on crop insect pests (Lee et al. 2020, Ramadan et al. 2020). Conversely, none of the release rates of thymol and eucalyptol elicited a disruptive effect on the bait-mediated attraction. Both thymol and eucalyptol show relevant bioactivity on *D. suzukii* as repellent and oviposition deterrent respectively (Erland et al. 2015, Renkema et al. 2016). In the case of thymol, an isomer of carvacrol, no repellency was detected, even when released from cotton discs at a release rate of approximately 15 mg/d, three times higher than that reported for carvacrol in the cellulose-containing Ziploc bags. Similarly, eucalyptol did not interrupt the attraction to the traps, despite being released at approximately five times the rate of carvacrol. Nevertheless, it should be pointed out that our assay represents a preliminary field test and, consequently, additional research would be required to fully address the feasibility of carvacrol as an effective repellent of *P. variegata* under natural conditions.

In spite of the availability of potential primary hosts (e.g., red foxes) in the sampling area, the oriental eyeworm *T. callipaeda* was not detected neither by dissection nor by PCR amplification in any of the pools of male flies examined. Nevertheless, the absence of the parasite in our samples should not be striking, as the infection rate under natural conditions has been reported to be very low. Indeed, only 1.34% of feral males were detected harboring *T. callipaeda* larval stages in a long-term (from April to November) and exhaustive (969 netted individuals of both sexes) fly collection conducted in southern Italy (Otranto et al. 2006b). For instance, and even though infestations by *T. callipaeda* in red foxes are known to occur in different countries with varying degrees of prevalence (Otranto et al. 2009, Hodžić et al. 2014, Čabanová et al. 2018, Ionică et al. 2018), the role of this wild carnivore species as a reservoir of the parasite in wildlife remains unknown. It is also noteworthy that so far, no autochthonous *T. callipaeda* infestations have been found in dogs in the Basque Country, with only five records of imported cases occurring since 2014 (Marino et al. 2020).

Taken together, these results represent a better understanding of the chemical ecology of *P. variegata,* providing a preliminary basis for the development of a promising management tool for *P. variegata* based on an easily customized trap and a food bait. In addition, our results suggest carvacrol as a potential repellent compound, deserving thus future in-depth research to unravel its applicability against *P. variegata*. In this regard, not only the use of an effective bait, but also the inclusion of a strong repellent, could be considered as promising tools for the control of *P. variegata* under a push–pull management strategy. The achievement of this goal would substantially contribute to develop a preventive measure against the spread of thelaziosis disease.
